# MicroRNA Profiling as a Novel Tool in the Diagnostics of Late-Onset Neonatal Sepsis: A Scoping Review

**DOI:** 10.3390/children12111573

**Published:** 2025-11-19

**Authors:** Eleni Papachatzi, Eleni Gkouti, Adamantia Kouvela, Sofia Benou, Gabriel Dimitriou, Sotiris Fouzas, Vassiliki Stamatopoulou, Despoina Gkentzi

**Affiliations:** 1 Neonatal Intensive Care Unit, University General Hospital of Patras, 26504 Patras, Greece; gdim@upatras.gr; 2 Department of Paediatrics, University General Hospital of Patras, 26504 Patras, Greece; up1105770@upatras.gr (E.G.); med6414@ac.upatras.gr (S.B.); sfouzas@upatras.gr (S.F.); gkentzid@upatras.gr (D.G.); 3 Department of Biochemistry, School of Medicine, University of Patras, 26504 Patras, Greece; kouvela.a@upatras.gr (A.K.); v.stam@upatras.gr (V.S.)

**Keywords:** microRNA, late-onset sepsis, biomarker, neonates

## Abstract

**Highlights:**

**What are the main findings?**

- Many studies show altered expression of miRNAs in septic neonates compared to controls.
- Existing studies are heterogenous, showing either up- or downregulation of specific miRNAs.

**What are the implications of the main findings?**

- MicroRNAs as novel biomarkers could play an important role in prompt and accurate diagnosis of late-onset sepsis (LOS), offering neonatologists a valuable tool.
- These findings require validation in larger cohorts and/or randomized control trials to determine their clinical utility.

**Abstract:**

**Background/Objectives:** The incidence of late-onset sepsis (LOS) has increased with improved survival rates in premature infants. Blood culture, the diagnostic “gold standard”, requires at least 36–72 h for results, leading to empiric antibiotic use and potential resistance. MicroRNAs (miRNAs) have emerged as promising biomarkers for sepsis in adults, but their role in neonatal LOS remains unclear. The aim of this scoping review is to identify the miRNA expression profiles of bacterial LOS in neonates. **Methods:** A scoping review of the literature was performed between 1 November 2023 and 31 December 2024. **Results:** Twelve studies fulfilled our criteria and were included in the review. Despite the considerable heterogeneity among the studies, most focused on detecting and quantifying serum microRNAs using real-time PCR, while some examined correlations with other biomarkers, such as CRP. The few microRNAs identified as common across multiple studies showed similar patterns of regulation in LOS cases. Compared to controls (no LOS), neonates with LOS exhibited significant alterations in miRNA expression. More precisely, in LOS, miRNA-181a, miRNA-23b, miRNA-181b5p, miRNA-21-5p, miRNA-34a5p, miRNA-199a3p, miRNA-1184 and miRNA-1295p were downregulated, while miRNA-16, miRNA-146a, miRNA-101, miRNA-187, miRNA-21, miRNA-15a/16 and miRNA-455-5p were upregulated. **Conclusions:** Currently, there is limited data regarding miRNA expression in LOS. Many studies showed altered expression of specific miRNAs in septic neonates; however, these observations need further validation in larger cohorts and/or randomized controlled trials to confirm their diagnostic potential.

## 1. Introduction

Despite significant advances in perinatal care, neonatal sepsis remains a leading cause of mortality, with an estimated rate of over 400.000 annual deaths worldwide [1]. The incidence of late-onset sepsis (LOS), above 72 h of life, has increased along with the improved survival rates of premature infants, highlighting the role of prolonged hospitalization in its pathogenesis. LOS remains a life-threatening condition with adverse outcomes for either term or preterm neonates [2]. The incidence of LOS varies geographically and is between 0.6 and 14%. The incidence varies across different gestational ages, from 1.6% in term neonates to 12–50% in very preterm or very-low-birth-weight neonates (VLBW) [2,3]. Although the clinical presentation of LOS is often non-specific, early and accurate diagnosis is of paramount importance to ensure prompt initiation of empiric treatment. On the other hand, over-consumption of antibiotics has led to the emergence of multi-resistant pathogens and long-term complications [4]. Pathogen isolation in blood culture, the current diagnostic gold standard, has several limitations, such as false negative results (low-level bacteremia, collection of small blood volume, previous antibiotic use) and false positive results (contamination with own flora), and requires 36–72 h of incubation [5,6,7]. The above reasons highlight the need for faster diagnostic methods with greater accuracy and efficiency. Pathogen identification by polymerase chain reaction offers high sensitivity; however, it cannot replace blood culture [8]. Additionally, numerous biomarkers (CRP, PCT, IL6, IL8) have been evaluated for LOS diagnosis, but none meet the essential criteria to replace current gold-standard methods [9,10]. Presepsin, a product of cleavage of CD14, is a promising biomarker in the diagnosis of sepsis. It is involved in the immune response (pathogen recognition and bacterial phagocytosis) [11]. In adult sepsis, presepsin has the potential to be used in clinical practice to aid in the diagnosis of severe bacterial infections and to predict unfavorable outcomes [12]. When combined with CRP, IL6 or PCT, it could be used as a diagnostic tool for neonatal sepsis; however, its role as a unique biomarker has not been clarified in the literature [13,14,15].

An ideal biomarker for LOS would facilitate early and accurate diagnosis of culture-confirmed LOS, could be used for all gestational ages and would have a rapid turnaround time. However, such a biomarker has yet to be identified [2]. MicroRNAs (miRNAs) are small (20–24 nucleotides), single-stranded RNA molecules that do not encode for proteins but play a crucial role in gene expression regulation. They were first described in 1993 by the research groups of Victor Ambros and Gary Ruvkun in the nematode Caenorhabditis elegans [16,17]. In 2024, both authors were awarded the Nobel Prize in Physiology or Medicine for their discovery of miRNAs and their involvement in post-transcriptional gene expression regulation [18]. Since their discovery, miRNAs have been identified in most eukaryotic organisms, with more than 2.300 miRNAs described in humans [19]. MiRNAs represent 1–5% of the human genome and regulate the expression of more than 60% of human genes [20].

MiRNAs are derived from individual genes, which are located in intergenic regions, introns or exons of genes encoding proteins [21]. They are transcribed primarily by RNA polymerase II and less frequently by RNA polymerase III, resulting in primary miRNA (pri-miRNAs) transcripts with a length ranging from 500 to 3000 nucleotides. The pri-miRNA is then cleaved by Drosha ribonuclease III and the DGCR8 protein, generating a precursor miRNA (pre-miRNA) of 70–80 nucleotides. The pre-miRNA is then transported to the cytoplasm via Exportin-5, where it is further processed by the Dicer endoribonuclease and forms a double-stranded RNA, 17–24 nucleotides long (miRNA duplex). Once the duplex is unwound, the mature miRNA is integrated into the RNA-induced silencing complex (RISC), where it binds to the target mRNA through sequence complementarity, leading to inhibition of translation [22,23].

As demonstrated by numerous studies, miRNAs are detectable in serum, plasma and a variety of other body fluids, a property that enables their use as non-invasive biomarkers. Unlike most circulating RNAs, which are typically unstable and rapidly degraded, miRNAs exhibit remarkable stability. This stability is primarily attributed to their binding to lipid transporters such as exosomes, microvesicles, apoptotic bodies, microparticles and protein complexes. Importantly, extracellular miRNAs remain biologically active and can modulate gene expression in target cells, acting as mediators of intercellular communication [24,25]. MiRNAs are best known for downregulating gene expression by targeting mRNAs for degradation or translation repression. However, they have also been reported to enhance the translation of specific genes under certain contexts, reflecting a more nuanced regulatory role than initially appreciated [26]. In addition to their stability, other important features make miRNAs ideal as biological indicators or diagnostic tools. These include their small size, their simple chemical structure and the lack of extensive post-transcriptional modifications [27,28].

MiRNAs are involved in various homeostatic pathways within the cell. Consequently, their deregulation has been involved in the pathogenesis of numerous diseases, including diabetes [27], cardiovascular disease and atherosclerosis [29,30,31], liver disease [32,33], cancer [34,35,36,37] and other diseases. Numerous studies have focused on changes in the expression of miRNAs caused by sepsis in adult populations. Some of the deregulated miRNAs that have been identified are miR-378a-3p, miR-193a-5p, miR-542-3p, miRNA-133a, miRNA-146a-5p, miRNA-150, miRNA-519c-5p, miRNA-3622b-3p, miRNA-122, miRNA-143, miRNA-155 and miRNA-192, among others [38,39,40,41,42]. Several of these miRNAs have been associated with the stage of disease and the prognosis of patients. The work of Roderburg C. et al., published in the *Journal of Clinical Medicine* in 2019, proposed a prognostic scoring system based on three miRNAs (miRNA-133a, miRNA-143 and miRNA-223), which, in combination with the age of the patient, can predict prognosis during hospitalization in the Neonatal Intensive Care Unit (NICU). Additionally, a separate score using two miRNAs (miRNA-133a and miRNA-150) combined with the age of the patient was found to predict long-term prognosis [28].

Several studies have highlighted the significance of miRNAs as biomarkers for diagnosing adult sepsis, and miRNAs nowadays emerge as critical regulatory elements in the pathogenesis of sepsis in adults [39,40,41]. On the other hand, there is limited data on their role in diagnosing neonatal sepsis, especially late-onset neonatal sepsis (LOS). The aim of this scoping review is to identify, collect and present a potential miRNA profile that is related to LOS in the neonatal population based on the published literature. As currently published research studies show considerable heterogeneity, this review aims to summarize the current knowledge and highlight future research directions regarding potential novel biomarkers (the miRNA expression pool) in the diagnosis of neonatal LOS.

## 2. Materials and Methods

This scoping review includes published original data reporting any association between miRNAs and late-onset neonatal sepsis. A literature review was performed from 1 November 2023 to 31 December 2024 in online databases (PubMed and SCOPUS). The literature search was conducted according to the PICO framework and was restricted to articles in English. The following keywords (and Boolean operators) were used: neonatal sepsis AND (miRNAs OR MicroRNAs) “late onset neonatal sepsis” AND/OR “micro-RNA” AND/OR “biomarkers” and the same keywords with no combination (“microRNAs”, “miRNAs”, “biomarkers”, “late onset sepsis”, “LOS”, “novel tool biomarker”, “neonatal sepsis”). Snowball searching was performed to search for further relevant articles in the reference list of included articles (forward and reverse citation tracking). The Preferred Reporting Items for Systematic Reviews and Meta-Analyses (PRISMA) guidelines were followed in this study in the study selection process [43]. At this point, the authors would like to highlight that even in the absence of a formal systematic review protocol, the PRISMA flow chart was used to transparently illustrate the process of literature identification, screening and selection.

### 2.1. Eligibility Criteria

Two unblinded reviewers screened all the relevant studies to determine whether it met the eligibility criteria. Any disagreements were discussed with a third reviewer. The eligibility criteria included all the available published studies reporting any association between miRNAs and late-onset sepsis in neonates written in the English language. These neonates were either already hospitalized in the NICU or pediatric ward and developed LOS or were admitted from the community with LOS. The definition of LOS was a systemic neonatal infection (with a positive blood culture) at 72 h of age or later (within the first 28 days of life).

### 2.2. Exclusion Criteria

Studies focusing on populations other than neonates (adults, children) were excluded from this study. Additionally, studies focusing on early-onset sepsis exclusively were excluded. If neonatal sepsis was not defined (early- or late-onset), these data were not included in the analysis. Moreover, studies focusing on miRNAs and their uses in other health conditions were not included in this review.

### 2.3. Selection Process

The abstract and the title of the relevant studies were reviewed by two reviewers for eligibility. Finally, the full texts of papers meeting the inclusion criteria and none of the exclusion criteria were reviewed by one reviewer and included in the analysis.

### 2.4. Generalizability and Transferability

The results included in this review have limited generalizability and transferability due to the heterogeneity of the studied populations (gestational age, day of life of LOS, comorbidities, ethnicity, and LOS definition, although all cases were culture-positive) and variations in the specific miRNA molecules/profile analyzed in each study. It is worth mentioning that most of the studies are of Asian/African origin and there is lack of data in the European population. Additionally, the sample sizes are relatively small, with all the studies using retrospectively collected data; moreover, statistical analyses differ among the included studies, limiting comparability. Moreover, most studies include healthy neonates as controls, except those of Wang et al. and Li et al., which include neonates with lower respiratory tract infection or pneumonia as controls. This raises uncertainty regarding whether the expressed miRNA profile is specific to sepsis or reflects a broader inflammation response. For this reason, future studies should include other inflammatory conditions in their cohorts, such as systemic inflammatory response syndrome (SIRS), congenital infections, necrotizing enterocolitis, intrauterine growth restriction and associated comorbidities, to better delineate miRNA expression patterns, etc.

## 3. Results

### 3.1. Study Selection

**Figure 1:**
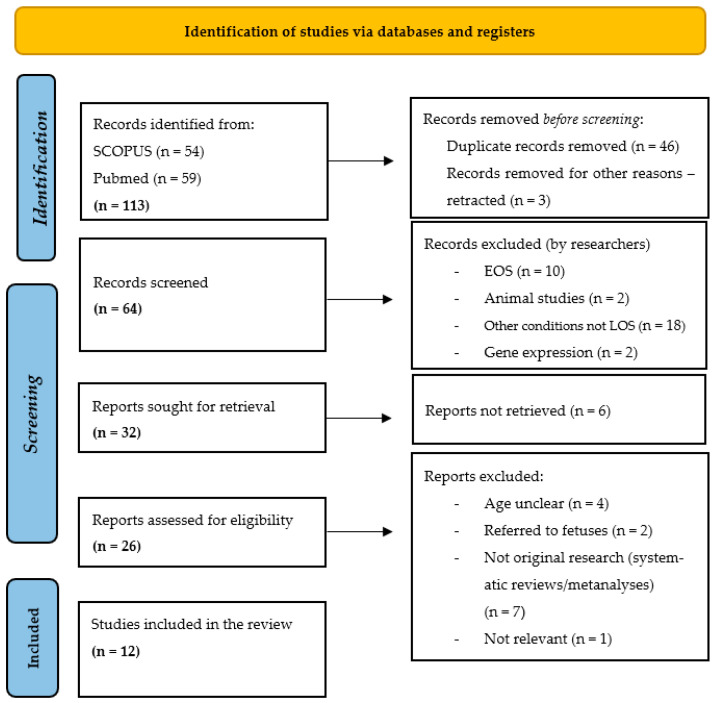
PRISMA 2020 flow chart for study selection [43].

A total of 113 relevant studies were identified in the databases (59 in PubMed and 54 in SCOPUS). Forty-six studies were duplicates and three were retracted. Of the remaining 64 studies, 32 were excluded as they referred to early-onset sepsis (EOS), were animal studies, or referred to other conditions unrelated to LOS and gene expression. The remaining 32 studies were sought for retrieval, but 6 were not available online [44,45,46,47,48,49]. Hence, 26 studies were assessed for eligibility, and finally, 12 studies met our criteria and were included in the analysis. All eligible studies were published between 2015 and 2024. The PRISMA flowchart is shown in Figure 1.

### 3.2. MicroRNA Profile

Most of the studies were retrospective, and their main characteristics are shown in Table 1. In 2015, in China, Wang et al. studied the expression of miRNA-15a, miRNA-16, miRNA-15b and miRNA-223 and the possible use of miRNA-15a/16 as a biomarker to predict neonatal sepsis outcome [50]. The researchers compared 46 neonates with sepsis (mean age of 12.64 ± 7.62 days) to 41 neonates with respiratory infection/pneumonia, who served as the control group. The authors demonstrated that the level of miRNA-15a/16 was upregulated in neonates with LOS compared to controls. No statistically significant differences in miRNA-15b and miRNA-223 levels were observed between the two groups. ROC analysis indicated miRNA-15a and miRNA-16 as potent biomarkers for the diagnosis of neonatal sepsis. The same team, in 2021, in a population of 66 neonates with LOS and 56 neonates with respiratory infection/pneumonia, found decreased serum levels of miRNA-1184 in the LOS group. Additionally, miRNA-1184 had a high diagnostic value in distinguishing LOS from respiratory conditions in neonates [51].

In the study by Ahmed et al., in 2017, serum miRNA-146a levels were measured in 24 septic neonates aged 8 to 17 days, 22 neonates at high risk for sepsis aged 8 to 18 days and 17 healthy controls aged 7 to 17 days. This age range indicates that the study specifically focused on LOS. The study revealed differential expression of miRNA-146a among the groups, with the highest levels observed in the sepsis group and the lowest in healthy controls. MiRNA-146a showed a significant positive correlation with CRP levels in the sepsis group [52].

A few years later, in 2020, Salim et al. studied the expression of miRNA-187, miRNA-101 and miRNA-21 as a potential diagnostic and prognostic tool for LOS. The authors included 50 neonates with LOS, 30 neonates with SIRS (and negative blood culture) and 20 healthy controls. Of note, miRNA-187 and miRNA-101 expression was increased in septic neonates compared to those with SIRS and healthy controls. In addition, expression levels of miRNA-21 were significantly higher in non-survivors compared to survivors [53].

During the same period, in 2020, in China, Liu et al. compared the miRNA-181a expression between 102 neonates with LOS (mean age of 11.52 ± 4.01 days) and 50 healthy controls and found significantly downregulated levels in the sepsis group (*p* < 0.01). Regarding the ROC curve, miRNA-181a had an AUC of miRNA 0.893, with a sensitivity of 83.3% and a specificity 84.0% [54]. In addition, in 2020, Fatmi et al., in Algeria, examined miRNA-23b expression in 48 neonates with sepsis, including 27 with EOS and 21 with LOS. MiRNA-23b levels were significantly reduced in both deceased and surviving LOS neonates with positive blood culture (*p* < 0.005 and *p* < 0.05, respectively). Notably, miRNA-23b was significantly lower in neonates who died of early-onset sepsis (*p* < 0.0001 in term infants, *p* < 0.05 in preterm), while it was increased in surviving EOS neonates with positive blood cultures (*p* < 0.005, *p* < 0.001) [55]. In another study conducted in China in 2020, Li et al. studied the expression of serum miRNA-129-5p in 75 LOS patients (mean age of 11.28 ± 4.59 days) and 84 controls without sepsis (but with respiratory infection or pneumonia). MiRNA-129-5p expression was significantly reduced in the sepsis group with a diagnostic value for sepsis, showing a sensitivity of 82.7% and a specificity of 79.8% [56].

More recently, in 2021, Mao et al. in China showed upregulated expression of serum miRNA-455-5p in neonates with LOS compared to controls (*p* < 0.001, AUC = 0.895). Their study included 99 neonates with LOS (mean age of 10.21 ± 4.04 days) and 88 healthy controls. Survival analysis indicated that high miRNA-455-5p expression was related to poor prognosis (log rank *p* = 0.015) [57]. In the same year, Serna et al., in Spain, found an alternate miRNomic expression in VLBW with Gram-positive LOS (compared to healthy controls). In detail, in their study, the miRNA profiles of 11 VLBW neonates with LOS were compared to those of 16 healthy controls. The authors found 33 miRNAs involved in gene expression regulation and immune and inflammatory responses. Their analysis revealed 217 differentially expressed miRNAs (*p* < 0.01); of those, 168 were upregulated (77.42%) and 49 downregulated (22.58%) in the sepsis group. Among these, 33 miRNAs were identified as potential candidates for gene expression regulation in a combined analysis [58].

In 2022, in a study in Egypt, Abdelaleem et al. compared the expression of serum miRNA-34a-5p and miRNA-199a-3p in 90 septic neonates (76 were blood culture-positive, 14 were -negative; 53 had EOS, and 37 had LOS) and 90 healthy controls. Both miRNA-34A-5p and miRNA-199A-3p levels were decreased in septic neonates compared to controls (*p* = 0.006, *p* = 0.001, respectively). Additionally, both miRNAs were significantly downregulated in neonates with positive blood culture, with miRNA-34a-5p found to be significantly lower in cases of Gram-negative bacterial sepsis (*p* < 0.0001), while miRNA-199a-3p was significantly decreased in Gram-positive bacteriemia (*p* < 0.0001). Τhere was no statistically significant difference in miRNA-34a-5p and miRNA-199a-3p expression between neonates with LOS and neonates with EOS (*p* = 0.307 and *p* = 0.912, respectively) [59]. In another study published in the same year in Egypt, Ali et al. examined the miRNA profiles of 60 neonates with sepsis (26 with EOS and 34 with LOS) and 60 healthy controls. miRNA181b-5p and miRNA21-5p were found to be significantly downregulated in the sepsis group (*p* < 0.001). Τhere was no statistically significant difference in the expression of the remaining miRNAs between neonates with LOS and neonates with EOS. Additionally, both miRNA181b-5p and miRNA21-5p miRNA were positively correlated to the SNAP II score and were significantly higher in non-survivors than survivors [60].

Finally, in the most recent study included in the present analysis, Katta et al., from India, reviewed miRNA expression in 50 neonates with LOS and 50 healthy controls. Their findings showed that miRNA-181a and miRNA-23b expression levels were significantly downregulated (*p* < 0.001) in LOS, with area under the curve (AUC) values of 0.83 and 0.92, respectively, whereas miRNA-16 expression was significantly upregulated (*p* < 0.001; AUC = 0.97). When compared with CRP levels, CRP had an AUC value of 0.831 (*p* < 0.001). Among the markers assessed, miRNA-23b showed the highest sensitivity (98%), whereas miRNA-16 exhibited the highest specificity (96%) [61].

## 4. Discussion

MiRNAs are small, non-coding RNA molecules that play a crucial role in gene expression regulation, influencing over 60% of human genes. They are involved in various diseases, including diabetes, cardiovascular disease, liver disease, cancer, and sepsis. In sepsis, specific miRNAs such as miRNA-25, miRNA-133a, miRNA-146a, miRNA-150, miRNA-223 and miRNA-192 have emerged as key regulators of the inflammatory response and potential biomarkers for predictive scoring [38,62,63,64,65,66,67]. MiRNA targets have a high potential for diagnosing pediatric sepsis; however, clinical impact is limited due to study heterogeneity and absence of reliable diagnostic robustness [15].

This review focuses on altered miRNA expression in neonatal LOS. In the neonatal population, miRNAs could serve as a rapid diagnostic tool with high sensitivity and specificity for distinguishing sepsis from non-sepsis cases [2]. This is of critical importance, as sepsis outcomes depend on prompt diagnosis and treatment. Sensitivity and specificity for miRNAs in sepsis have been analyzed for both adults and children/neonates [68,69]. To date, numerous miRNAs, such as miRNA-146a, miRNA-223 and miRNA-155-5p, have emerged as promising biomarkers for sepsis [70]. According to the results of a meta-analysis using a random-effects model, C. Ren et al. described the pooled specificity and sensitivity of specific miRNAs (miRNA-96, miRNA-34a-5p, miRNA-199a-3p, miRNA-15b, miRNA-378a, miRNA-1184, miRNA-16a, miRNA-451, miRNA-141, miRNA-455-5p, miRNA-129-5p, miRNA-181a, miRNA-15a, miRNA-16, miRNA-21, miRNA-29a, miRNA-101-3p, miRNA-211-5p, miRNA-142-3p, miRNA-101 and miRNA-187) for the diagnosis of neonatal sepsis, which were 0.83 (95% CI: 0.79–0.87) and 0.76 (95% CI: 0.72–0.80), respectively. Their findings align with those of Y. Qiu et al., who reported pooled specificity and sensitivity values of miRNA of 0.85 (95% CI 0.80–0.89) and 0.80 (95% CI 0.75–0.83), respectively. Additionally, the overall accuracy value of miRNAs (79.02%) was significantly higher than that of CRP (61.22%) (*p* < 0.000) [68,69].

Our review showed that the expression of miRNAs in LOS was either up- or downregulated compared to healthy controls or neonates with respiratory infection/pneumonia (*). More precisely, miRNA-181a, miRNA-23b, miRNA-181b5p, miRNA21-5p, miRNA-34a5p, miRNA-199a3p, miRNA-1184 (*) and miRNA-1295p (*) were downregulated in neonates with LOS, while miRNA-16, miRNA-146a, miRNA-101, miRNA-187, miRNA-21, miRNA-15a/16 (*) and miRNA-455-5p were upregulated compared to controls. Consistency in the results, among different centers, was found for miRNA-23b and miRNA-181a, which were downregulated, as well as for miRNA-16, which was upregulated.

Regarding sampling and laboratory analyses, most studies used serum for miRNA isolation and differential expression analysis, while two studies used whole blood, and only one specifically examined the monocyte fraction. Notably, besides one single study that analyzed the miRNA expression profile using microarrays, all others focused on specific miRNAs through real-time quantitative PCR (RT-qPCR). This highlights the need for higher-throughput approaches, such as next-generation RNA sequencing, to further investigate the role and the diagnostic and prognostic capabilities of miRNAs in LOS, leading to the identification of potentially novel biomarkers.

Regarding miRNA use as a diagnostic tool for LOS, area under the curve (AUC) values were presented in only 3 out of 12 studies. The downregulation of miRNA-181a and miRNA-23b had AUC values of 0.83 and 0.92, respectively, whereas miRNA-16, which was significantly upregulated (*p* < 0.001), had an AUC of 0.97 [61]. Additionally, the upregulated expression of serum miRNA-455-5p (*p* < 0.001) had an AUC of 0.895. Moreover, miRNA-455-5p level was positively correlated with white blood cell count and other clinical characteristics (*p* < 0.01) [57]. In the study of Liu et al., miRNA-181a was found to be significantly downregulated in the sepsis group (*p* < 0.01), with an AUC of 0.893, a sensitivity of 83.3% and a specificity of 84.0% [42].

Concerning miRNA expression and its possible relation to neonatal prognosis in LOS, only 4 out of 12 studies included relevant data. Mao et al. showed that high miRNA-455-5p expression was related to poor prognosis (log rank *p* = 0.015) [57]. Salim et al. found that the expression of miRNA-21 was significantly higher in non-survivors compared to survivors [53]. Likewise, Fatmi et al. found that miRNA-23b expression was decreased in LOS, with significantly lowered levels in the fatal sepsis cases (*p* < 0.05). Ιn addition, miRNA-23b expression exhibited a positive correlation with the number of survivors in LOS (correlation coefficient = 0.70, *p*  =  0.506) [55]. In the study of Ali et al., miRNA-181b-5p and miRNA-21-5p, which were significantly downregulated in the sepsis group (*p* < 0.001), were also significantly higher in non-survivors compared to survivors [60].

Although this review presents published data regarding miRNA expression in neonatal sepsis, most of the studies did not provide a receiver operating characteristic analysis (ROC) for their findings. As a result, it is rather difficult and remains challenging to conclude whether these miRNAs could act as reliable biomarkers for LOS. Additionally, taking into consideration the variability in reported miRNAs described in each study, the results could not be directly compared or validated across different centers. Towards this direction and given the heterogeneity and inconsistency of the data, a meta-analysis would likely yield inconclusive results at this stage. However, the results presented herein, and especially the specific miRNAs with altered expression, underscore the need for further prospective studies in larger neonatal population samples. Researchers should focus not only on identifying novel biomarkers but also assessing the expression of the already known miRNAs related to LOS-miRNA. There is growing and promising evidence that miRNAs could be ideal biomarkers for neonatal LOS, and future research in the field is anticipated to enhance our understanding of sepsis pathogenesis in this vulnerable population [71].

## 5. Conclusions

There is limited data on miRNA’s role in the early diagnosis of neonatal LOS, especially in the European population. The identification of a specific miRNA profile for LOS could offer neonatologists a valuable tool for early diagnosis and targeted treatment, which are both crucial in the setting of the Neonatal Intensive Care Unit. To this end, prompt and accurate diagnosis of LOS using novel biomarkers could also help decrease unnecessary antimicrobial use, minimizing the risk of the emergence of multi-resistant pathogens and antibiotic-related complications. Existing studies are heterogenous, showing either up- or downregulation of specific miRNAs. However, these observations require validation in larger cohorts and/or randomized control trials to determine their clinical utility. MiRNAs show considerable promise as novel biomarkers for diagnosing LOS. However, current evidence is insufficient to support their clinical application. Further research is needed to clarify their utility and potential impact on the management of neonatal sepsis [39].

## Figures and Tables

**Table 1:** Studies included in this review, date of publication, authors, sample population and main findings regarding miRNA profile.

Study Title and Author	Year	Origin	Population	Used for miRNA Extraction	MiRNA Profile/Result Upregulated (U) Downregulated (D)
miRNA-15a/16 are upregulated in the serum of neonatal sepsis patients and inhibit the LPS-induced inflammatory pathway, Wang et al. [50]	2015	China	46 neonates with sepsis and 41 neonates with respiratory infection/pneumonia	Serum	miRNA-15a/16 (U)
Downregulation of miRNA-1184 serves as a diagnostic biomarker in neonatal sepsis and regulates LPS-induced inflammatory response by inhibiting IL-16 in monocytes, Wang et al. [51]	2021	China	72 neonates with neonatal sepsis (including 6 cases of EOS and 66 cases of LOS) and 56 neonates with respiratory infection or pneumonia	Serum	miRNA-1184 (D)
Value of Serum miRNA 146a as Biomarker for Early Diagnosis of Neonatal Sepsis. Ahmed et al. [52]	2017	Egypt	24 neonates with LOS, 22 neonates at high risk of sepsis and 17 healthy controls	Serum	miRNA-146a (U)
Evaluation of the clinical value of circulating miRNA-101, miRNA-187 and miRNA-21 in neonatal sepsis diagnosis and prognosis, Salim et al. [53]	2020	Egypt	100 neonates (50 with LOS with positive blood culture, 30 with SIRS with negative blood culture and 20 healthy neonates)	Serum	miRNA-101 (U), miRNA-187 (U), miRNA-21 (U)
Clinical significance of miRNA-181a, in patients with neonatal sepsis and its regulatory role in the lipopolysaccharide-induced inflammatory response, Liu et al. [54]	2020	China	102 cases with LOS and 50 healthy controls	Serum	miRNA-181a (D)
MiRNA-23b as a biomarker of culture-positive neonatal sepsis, Fatmi et al. [55]	2020	Algeria	48 neonates with sepsis (27 with EOS, 21 with LOS)	Serum	miRNA-23b (D)
Clinical significance of miRNA-129-5p in patients with neonatal sepsis and its regulatory role in the lipopolysaccharide-induced inflammatory response, Li et al. [56]	2020	China	75 newborns with neonatal sepsis and 84 newborns with respiratory infection or pneumonia but without a picture of sepsis	Serum	miRNA-129-5p (D)
Clinical significance of the serum miRNA-455-5p expression in patients with neonatal sepsis, Mao et al. [57]	2021	China	99 cases with LOS and 88 healthy controls	Serum	miRNA-455-5p (U)
MiRNomic signature in very low birth-weight neonates discriminates late-onset Gram-positive sepsis from controls, Serna et al. [58]	2021	Spain	11 very-low birth-weight neonates with late-onset Gram-positive sepsis and 16 controls	Not clear	miRNA-31-5p, miRNA-1271-5p, miRNA-326, miRNA-146b-5p, miRNA-140-5p, miRNA-409-5p, miRNA-668-3p, miRNA-27b-3p, miRNA-28-5p, miRNA-152-3p, miRNA-431-5p, miRNA-106b-5p, miRNA-151a-3p, miRNA-15a-5p, miRNA-339-5p, miRNA-30b-5p, miRNA-146a-5p, miRNA-20a-5p, miRNA-20b-5p, miRNA-532-5p, miRNA-30c-5p, miRNA-106a-5p, miRNA-17-5p, miRNA-23b-3p, miRNA-93-5p, miRNA-425-5p, miRNA-1298-5p, miRNA-107, miRNA-103a-3p, miRNA-372-3p, miRNA-760, miRNA-6088
Serum miRNA-34a-5p and miRNA-199a-3p as new biomarkers of neonatal sepsis, Abdelaleem et al. [59]	2022	Egypt	60 cases with LOS and 60 healthy controls	Serum	miRNA-34a-5p (D), miRNA-199a-3p (D)
Diagnostic and prognostic values of miRNA181b-5p and miRNA21-5p for neonatal sepsis risk and their link to SNAP II score and disease mortality, Ali et al. [60]	2023	Egypt	60 cases with LOS and 60 healthy controls	Serum	miRNA181b-5p (D), miRNA21-5p (D)
Sensitivity of miRNA-181a, miRNA-23b and miRNA-16 in the Late-Onset Neonatal Sepsis: A Diagnostic Study, Katta et al. [61]	2024	India	50 cases of culture-proven LOS and 50 healthy controls	Serum	miRNA-181a (D), miRNA-23b (D), miRNA-16 (U)

## Data Availability

The original contributions presented in the study are included in the article, further inquiries can be directed to the corresponding author.

## Abbreviations

The following abbreviations are used in this manuscript:

LOS	Late-onset sepsis
MiRNA	MicroRNA
PRISMA	Preferred Reporting Items for Systematic Reviews and Meta-Analyses
RISC	RNA-induced silencing complex (RISC)
CRP	C-reactive protein
PCT	Procalcitonin
IL6	Interleukin 6
IL8	Interleukin 8
RISC	RNA-induced silencing complex
RNA	Ribonucleic acid
PICO	Population, intervention, comparison, and outcome
SIRS	Systematic inflammatory response syndrome
EOS	Early-onset sepsis
ROC	Receiver operating characteristic
VLBW	Very low birth weight
SNAP II	Score for neonatal acute physiology II
AUC	Area under the curve
LPS	Lipopolysaccharide
PCR	Polymerase chain reaction
RT-qPCR	Quantitative reverse-transcription polymerase chain reaction
